# Efficacy and safety of Chinese medicines for vitreous hemorrhage: A protocol for systematic review and meta-analysis

**DOI:** 10.1097/MD.0000000000020086

**Published:** 2020-05-08

**Authors:** Mengyu Han, Ziqiang Liu, Luqi Nong, Yingxin Zi, Huan Meng, Yu Deng, Zhi-Jun Wang, Ming Jin

**Affiliations:** a Beijing University of Chinese Medicine; b Department of Ophthalmology, China-Japan Friendship Hospital, Beijing, China.

**Keywords:** protocol, systematic review, traditional Chinese medicines, vitreous hemorrhage

## Abstract

**Background:**

Vitreous hemorrhage (VH) is a common ophthalmic disease with a high rate of blindness, which will seriously affect the quality of life of patients and bring great burden to patients’ families and society. The treatment for VH contains medical therapy, lasers, and surgery. At present, there is no recognized western medicine with definite curative effect and little side effect for the treatment of VH. In most cases, PRP is not available to treat VH; intravitreal injection or surgical treatment is adopted as the primary therapy. However, in the long-term treatment, the effect of the above-mentioned treatment is not satisfactory, so many patients choose oral Chinese medicines, which has been widely used in China to treat VH. Numerous clinical trials have demonstrated that Chinese medicines can promote the absorption of VH and improve the visual function of patients. The purpose of this review is to evaluate the efficacy and safety of Chinese medicines in the treatment of VH and inform a decision aid for the clinical encounter between patients and clinicians. Besides, it is beneficial to establish a future research agenda.

**Methods:**

The systematic review will include all of the randomized controlled trials on the efficacy and safety of Chinese medicines for VH. Nine electronic databases, namely PubMed, Web of Science, EMBASE, the Cochrane Library, Google Scholar, China National Knowledge Infrastructure (CNKI), Wanfang Database, China Science and Technology Journal database (VIP), and CBM, will be searched normatively on the basis of the rule of each database from the inception to August 31, 2019. We will also search registers of clinical trials, potential gray literature, and conference abstracts. There are no limits on language and publication status. The literature screening, data extraction, and quality assessment will be conducted by 2 reviewers independently. The reporting quality and risk of bias will be assessed by other 2 researchers. Standard of curative effect and total treatment efficacy rate were assessed as the primary outcome. The secondary outcomes will include the curative effect of single symptom and sign, the improvement rate of single auxiliary examination, withdrawal and reduction of western medicines in a course of treatment, maintenance of western medicines after the course of treatment, laboratory efficacy indexes. Meta-analysis will be performed using RevMan5.3 software provided by the Cochrane Collaboration.

**Results:**

This study will provide a comprehensive review based on current evidence of Chinese medicines treatment for VH in several aspects, including standard of curative effect, total treatment efficacy rate, the curative effect of single symptom and sign, the improvement rate of single auxiliary examination, withdrawal and reduction of western medicines in a course of treatment, laboratory efficacy indexes, total treatment efficacy, and safety, among others.

**Conclusion:**

The conclusion of this study will provide evidence to determine whether Chinese medicines are an effective and safe intervention for patients with VH.

**Ethics and dissemination:**

It is not necessary to obtain ethical approval for this study. The systematic review will be published in a peer-reviewed journal, presented at conferences and will be shared on social media platforms.

**PROSPERO registration number:**

PROSPERO CRD42020152321.

## 1 Introduction

Vitreous hemorrhage (VH) is one of the common vitreoretinal diseases in ophthalmology. VH not only makes the refractive stroma cloudy, affecting the light to reach the retina, leading to vision loss, but also leads to vitreous degeneration, posterior detachment, and many serious complications such as macular edema, optic nerve atrophy, and so on.^[1]^ The causes of VH depend on the study population and vary with the mean age of the patients and the region where the study is performed. Fundus vascular diseases and ocular trauma, the leading cause in children and adolescents,^[2,3]^ are the most common causes of VH.^[4,5]^ The formation of retinal neovascularization (NV) plays a key role in the pathological mechanism of nontraumatic VH.^[6]^ Hypoxic ischemia of retinal tissue results in increased secretion of vascular endothelial growth factor (VEGF), insulin-like growth factor-1, and other cytokines.^[7,8]^ Such angiogenic factors are present in the vitreous and whole retina of patients,^[9]^ which further forms NV between retinal-vitreous, leading to a VH.^[10]^ In adults, proliferative diabetic retinopathy (PDR) and age-related macular degeneration are the most common causes of retinal NV.^[11]^ Diabetic retinopathy (DR), the chief cause of severe vision loss in adults of working age, worldwide,^[12]^ accounted for 50.0%∼65.0% of the causes of nontraumatic VH.^[13]^According to a recent survey of retina specialists, VH from PDR is one of the most common reasons for vitrectomy in the United States,^[14]^ which not only affects vision substantially but also can preclude performing panretinal photocoagulation (PRP), the standard treatment for PDR.^[15]^ China has the largest population of diabetic patients in the world, with >92 million diabetic patients older than 20 years.^[16]^ The prevalence of retinopathy in diabetic patients is up to 24.7% to 37.5%, among which the proportion of proliferative retinopathy is 3.3%∼7.4%.^[17]^ VH can be divided into 1 to 4 levels (Table 1).^[18,19]^

**Table 1:**
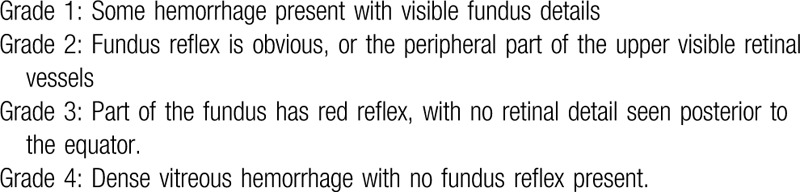
Grading of vitreous hemorrhage.

The treatment for VH contains medical therapy, lasers, and surgery. At present, there is no recognized western medicine with definite curative effect and little side effect for the treatment of VH. PRP is used for the treatment of mild to moderate cases of VH when the refractive stroma permits.^[20]^ However, in most cases, PRP is not available to treat VH; vitrectomy is the primary treatment. Although vitrectomy has the advantages of one-time removal of cloudy vitreous and rapid recovery of impaired visual function, it cannot solve the problems of vascular tension and blood density, at the same time, surgical complications that can be fatal to vision remain including neovascular glaucoma, retinal detachment, endophthalmitis.^[21]^ In addition, recovery to normal activities following vitrectomy can take several days, weeks, or even months, thus affecting an individual's ability to function and work.^[22]^Anti--VEGF treatment has also been used for VH in recent years,^[22]^ but currently there is no established guideline and its effect is ambiguous^[19]^ and NV tends to recur,^[23]^ and it may cause retinal atrophy,^[24]^ retinal pigment epithelium tears,^[25]^ systemic adverse effects,^[26]^ among others.

As a complementary and alternative therapy, Chinese medicines have a long history and great potential, which is widely used in the treatment of VH in China. The effectiveness of Chinese medicines in treating VH has been proved by many clinical and experimental studies. Samul-Tang significantly inhibited retinal NV by downregulating hypoxia inducible factor-1α (HIF-1α), stromal cell derived factor-1(SDF-1), CXCR4, and VEGF.^[27]^ Formononetin, an active compound of Astragalus membranaceus (Fisch) Bunge, can ameliorate retinal NV via the HIF-1α/VEGF signaling pathway, and it may become a potential drug for the prevention and treatment of DR.^[28]^ “Stasis sanjie” method can effectively improve the absorption degree of VH, reduce its complications, and restore the visual function of patients.^[29]^

Although the literature on Chinese medicines treatment of VH has increased rapidly in general, to our knowledge, there is a lack of critically designed systematic review to evaluate the effectiveness and safety of Chinese medicines for VH. In this study protocol, we will present the protocol and assess all of the clinical evidence on the effectiveness and safety of Chinese medicines for VH patients and inform a decision aid for the clinical encounter between patients and clinicians. Besides, it is beneficial to establish a future research agenda.

## 2 Methods

The systematic review protocol has been registered on PROSPERO (registration number: CRD42020152321).^[30]^ Our protocol will follow the Cochrane Handbook for Systematic Reviews of Interventions and the Preferred Reporting Items for Systematic Reviews and Meta-Analysis Protocol (PRISMA-P) statement guidelines.^[31,32]^

### 2.1 Inclusion criteria for study selection

#### 2.1.1 Types of studies

We will consider only clinical randomized controlled trials (RCTs) of Chinese medicines in the treatment of VH. The current clinical trial results will be objectively integrated, which is conducive to the evaluation of the efficacy and safety of Chinese medicines for VH. We will exclude non-RCTs, quasi- RCTs, uncontrolled trials, reviews, case reports, case-controlled studies, animal trials, and laboratory studies.

#### 2.1.2 Types of patients

Patients diagnosed as having VH will be included in the study. There will also be no restrictions based on other conditions, such as age, sex, race, educational or economic status, disease duration, and disease severity.

#### 2.1.3 Types of interventions

This study focuses on the RCTs of VH with the therapy of Chinese medicines, and the results will provide advice and consultation for clinicians. Therefore, patients in the experimental group were only treated with Chinese medicines, and the types and dosage forms of Chinese medicines prescriptions were not limited. In addition, western medicines and other treatment methods were not combined. Studies that with combination therapy fail to objectively evaluate the efficacy and safety of Chinese medicines will be excluded. The control interventions will include placebo, western medicines, surgery and other therapies.

#### 2.1.4 Types of outcome measures

##### 2.1.4.1 Primary outcomes

Standard of curative effect^[19]^ and total treatment efficacy rate were assessed as the primary outcome. There are 4 levels about standard of curative effect on the basis of the grade of VH (determined by slit-lamp examination and ophthalmoscopy, Table 1) and visual acuity (VA),which were noted at each follow-up (Recovery: VH is absorbed completely or basically, VA is restored before onset; Marked Effect: VH most absorption, leaving only a small amount of retinal spotting hemorrhage, improve vision more than 4 lines; Validity: VH partial absorption, improve VA 1∼3 lines; Invalidity: VH and VA are compared before treatment without apparent change, or VA drops). Total treatment efficacy = recovery rate+ marked effect rate + validity rate.

##### 2.1.4.2 Secondary outcomes

The secondary outcomes of this review mainly include the following aspects:

1. The curative effect of single symptom and sign: best corrected visual acuity (BCVA), and so on.
2. The improvement rate of single auxiliary examination: visual field examination, optical coherence tomography, and ultrasound B-scan.
3. Withdrawal and reduction of western medicines in a course of treatment, including: time, type and quantity; maintenance of western medicines after the course of treatment, including type and quantity.
4. Laboratory efficacy indexes: VEGF, IGF-1.

##### 2.1.4.3 Security index

The safety outcomes will be measured by the incidence and severity of side-effects. Any unexpected events that occurred during the studies will be recorded on an adverse event report form, including:

1. General physical examination (temperature, pulse, respiration, blood pressure)
2. Routine examination of blood, urine and stool
3. Liver and kidney function examination
4. Electrocardiogram
5. Possible complications (posterior subcapsular cataract, macular ischemia, rebleeding, tractional retinal detachment, tractional retinal break, retinal tear)
6. adverse reactions and related detection indicators.

### 2.2 Search methods for the identification of studies

#### 2.2.1 Electronic searches

Nine electronic databases, namely PubMed, Web of Science, EMBASE, the Cochrane Library, Google Scholar, China National Knowledge Infrastructure (CNKI), Wanfang Database, China Science and Technology Journal database (VIP)and CBM, will be searched normatively according to the rule of each database from the inception to August 31, 2019 for reviews on Chinese medicines and VH. The search term will include two parts: that is, “Medicine, Chinese Traditional” (eg, “Medicine, Chinese Traditional,” TCM, Traditional Chinese medicine, Zhong Yi Xue) and VH. The equivalent search entries will be used while searching in the Chinese databases. The search strategy for PubMed is listed in Table 2, which including all search terms, and other searches will be conducted based on these results. This will be appropriately adapted for search in the other databases.

**Table 2:**
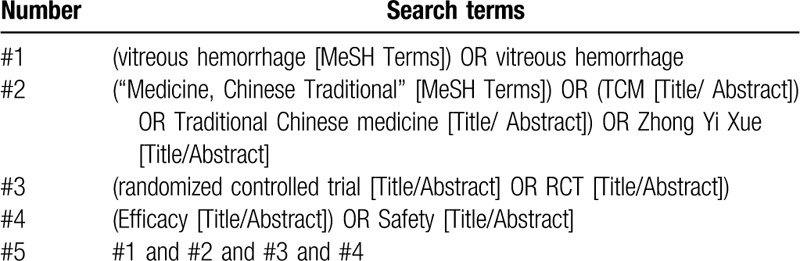
Search strategy used in PubMed database.

#### 2.2.2 Searching other resources

Meanwhile, we will also search PROSPERO, the International Clinical Trials Registry Platform (ICTRP), ClinicalTrials.gov, dissertations, and gray literature to identify systematic reviews or clinical trials related to Chinese medicines and VH. Relevant journals and conference processes will be manual searched. We will also review articles and bibliographies included in the trials.

### 2.3 Data collection and analysis

#### 2.3.1 Selection of studies

**Figure 1:**
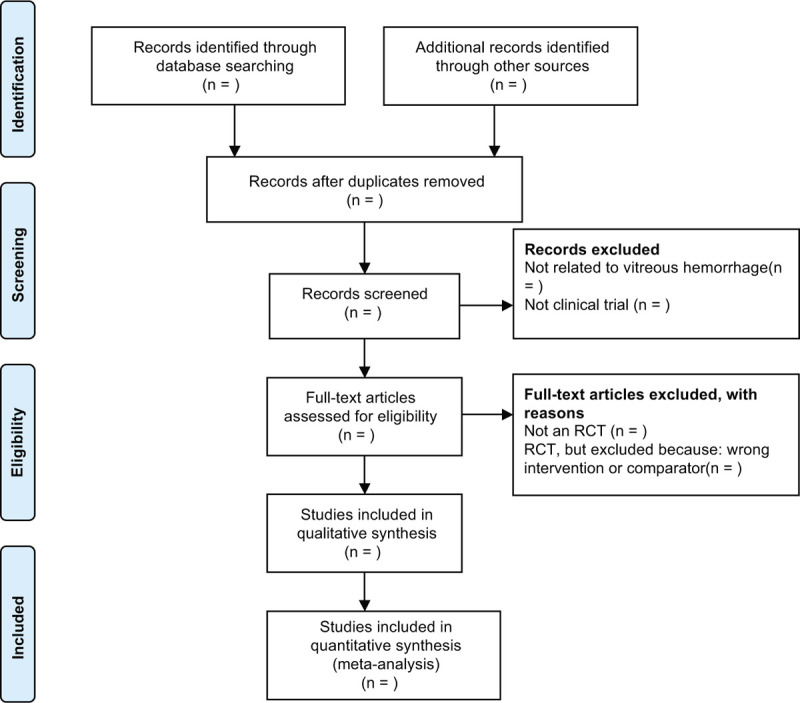
The PRISMA flow chart of the selection process. PRISMA = Preferred Reporting Items for Systematic Reviews and Meta-Analysis protocol.

We will select studies involved any form of Chinese medicines as the sole treatment or as a major therapy. Chinese medicines will be classed as the major therapy when the literature suggests that the frequency of application of Chinese medicines is higher and the time is longer than other intervention methods. Studies only related to human subjects will be included. Two reviewers (MYH and ZQL) will independently browse the titles, abstracts, and keywords of all of the retrieved records to distinguish and exclude any obviously irrelevant articles. If these reviewers have disagreements, a third author (MJ) will make the final decision. The study selection procedure is presented in a PRISMA flow chart (Fig. 1).

#### 2.3.2 Data extraction and management

Based on the inclusion criteria, a standard data collection form will be produced before data extraction. EndNote X8 software will be used to manage the records we obtained from electronic databases and other resources. Two authors (MYH and ZQL) will extract the data of interest from the eligible study and enter the following information in the data extraction sheet: The basic characteristics of each study (study type, author, title, source/journal, time of publication, country, hospital setting, study design); participants characteristics (average age, sex, sample size, inclusion and exclusion criteria, baseline situation); interventions (type of Chinese medicines, randomization, allocation concealment, blinding methods, and duration and frequency); comparators (western medicines, surgery); outcomes (measures, main outcomes, security indexes, and follow up); if funded, it will also be recorded. When the consensus on data extraction is not available through discussion, the third reviewer (MJ) will make a decision.

#### 2.3.3 Assessment of risk of bias

Two authors (LQN and ZQL) will independently evaluate the risk and bias using the Cochrane risk of bias (ROB) assessment tool.^[33]^ The RevMan software program (V.5.3) will record the selected details of each study.^[34]^

#### 2.3.4 Measures of treatment effect

The risk ratio (RR) and 95% confidence interval (CI) will be used to analyze dichotomous data and measure the treatment effect. A weighted mean difference (WMD) or a standard mean difference (SMD) with 95% CIs will be used to analyze continuous outcomes.

#### 2.3.5 Unit of analysis issue

We will only extract the 1^st^ experimental period data of crossover trials to avoid carryover effects. Meanwhile, considering that there are multiple intervention groups in trials, we will combine all analogous groups into a single pairwise comparison to prevent a unit of analysis issue.

#### 2.3.6 Management of missing data

If any details of the trial are incomplete, reviewer (HM) will contact the appropriate author of an article via email and telephone to obtain any missing data. The missing data will be deleted, if there is no response from the author. In this case, this will be addressed in the discussion.

#### 2.3.7 Assessment of heterogeneity and data synthesis

We will use the complete case data as the analysis data. Heterogeneity will be tested with a standard *χ*^2^ test.^[35]^ To quantify the impact of the statistical heterogeneity on the systematic review, the *I*^2^ value will be applied to calculate and present the heterogeneity degree. When *P* > .1, *I*^2^ < 50%, it is considered that there is no heterogeneity between the trials, and the fixed-effect model will be used; otherwise, the random-effect model will be adopted. Data analysis will be performed using RevMan5.3 software provided by the Cochrane Collaboration. Using the software to obtain forest plots and test the heterogeneity between the included studies. The Grades of Recommendation, Assessment, Development, and Evaluation (GRADE) will be used to assess the meta-analysis findings and describe the strength of evidence. Narrative comprehensive synthesis will be adopted, if meta-analysis is not possible due to lack of clinical studies or heterogeneity.

#### 2.3.8 Assessment of reporting biases

The funnel plot and statistic test will be adopted to evaluate reporting biases, when ≥10 studies are included in a meta-analysis.

#### 2.3.9 Subgroup analysis

When heterogeneity is detected, a subgroup analysis will be conducted to judge the source of heterogeneity. The criteria for a subgroup analysis are as follows:

1. Type of Chinese medicines therapies
2. Research quality
3. Participation population
4. Type of control interventions
5. Intervention frequency and duration.

#### 2.3.10 Sensitivity analysis

In the case of sufficient trials data, the ROB tool will be used to assess methodological quality. If low-quality articles are deleted, a second meta-analysis will be performed. The results and effect size of the 2 meta-analyses will be compared and discussed.^[36]^

## 3 Discussion

VH is one of the most common diseases in ophthalmology, which has destructive impact on patients’ VA and negative effect on their quality of life and social development. Treatment for VH mainly includes medication, lasers, and surgery. At present, there is no recognized western medicine with definite curative effect and little side effect for the treatment of VH. In addition, in most cases, PRP is not available to treat VH, vitrectomy is the primary treatment.^[20]^ But vitreous surgery equipment is expensive, the treatment cost is high, the technical level requirements are high, the basic hospital is not easy to carry out the operation, and vitrectomy has a series of complications, some patients even vitreous massive hemorrhage is not willing to receive surgery treatment. So how to treat the disease safely, cheaply, and effectively is an important topic. In today's world especially China, many patients choose oral Chinese medicines instead of other treatments. Chinese medicines have a certain unique therapeutic effect on VH. However, due to the complex composition of Chinese medicines, potential safety hazards may exist. The purpose of this systematic review is to systematically summarize and evaluate a great amount of evidences for Chinese medicines treatment for VH, and to evaluate the efficacy and safety of Chinese medicines in the treatment of VH and inform a decision aid for the clinical encounter between patients and clinicians. In addition, it helps to establish a future research agenda.

## Author contributions

**Conceptualization:** Mengyu Han, Ziqiang Liu, Zhijun Wang, Ming Jin.

**Data curation:** Mengyu Han, Ziqiang Liu, Luqi Nong, Huan Meng.

**Formal analysis:** Mengyu Han, Ziqiang Liu.

**Project administration:** Mengyu Han, Ziqiang Liu, Luqi Nong, Ming Jin.

**Supervision:** Mengyu Han, Zhijun Wang, Ming Jin.

**Writing – review & editing:** Mengyu Han, Ziqiang Liu, Luqi Nong.
